# Supplementary figures and images for: Min-redundancy and max-relevance multi-view feature selection for predicting ovarian cancer survival using multi-omics data
Source: BMC Med Genomics. 2018 Sep 14;11(Suppl 3):71. doi: 10.1186/s12920-018-0388-0 (PMC6157248; doi:10.1186/s12920-018-0388-0)

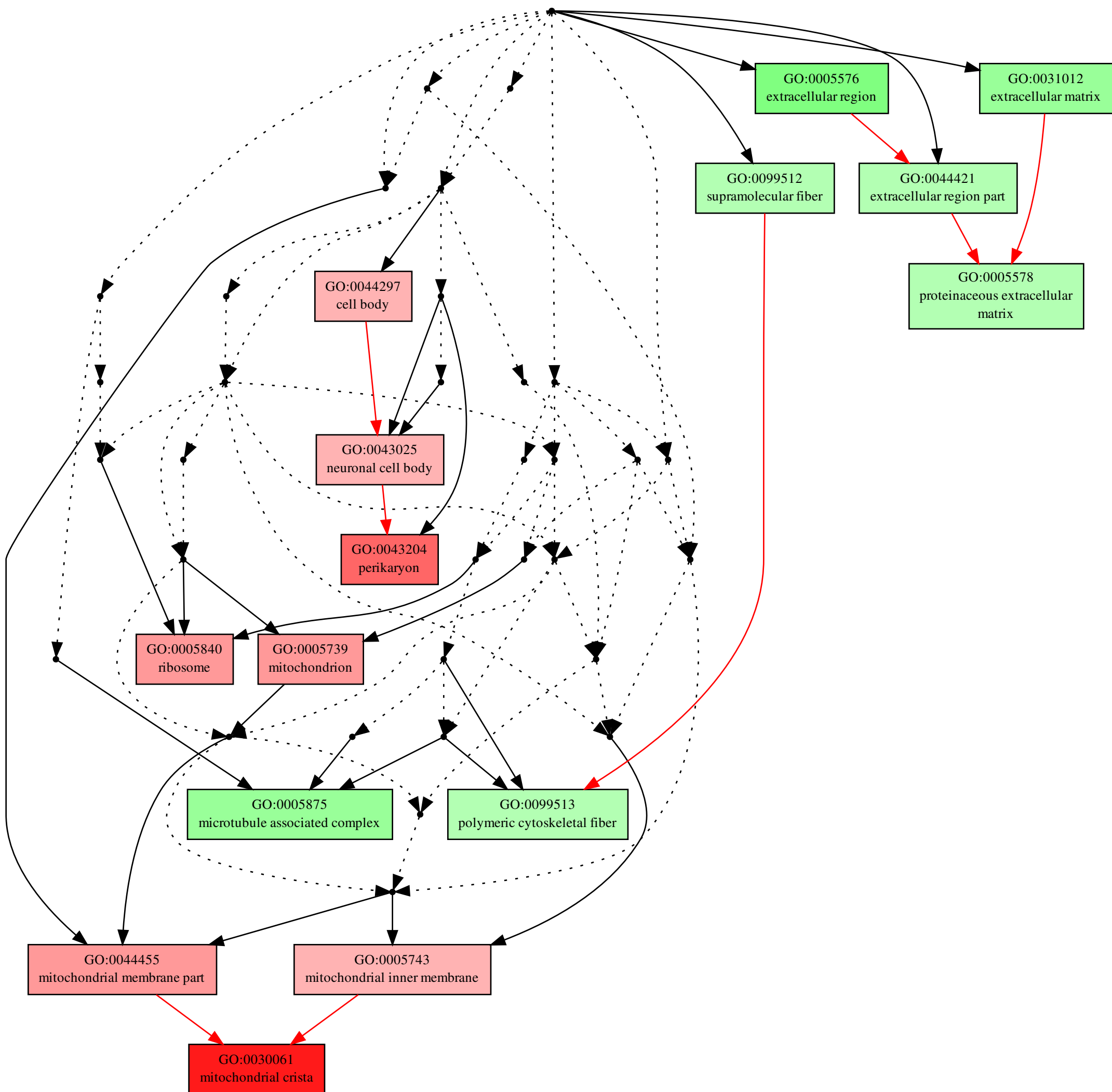

Supplement: Supplementary file 4 — Graphical output of Multi-GOEAST analysis results of Cellular Components GO terms in the top selected genes in CNA and RNA-Seq. (PDF 56 kb) [file 12920_2018_388_MOESM4_ESM.pdf]

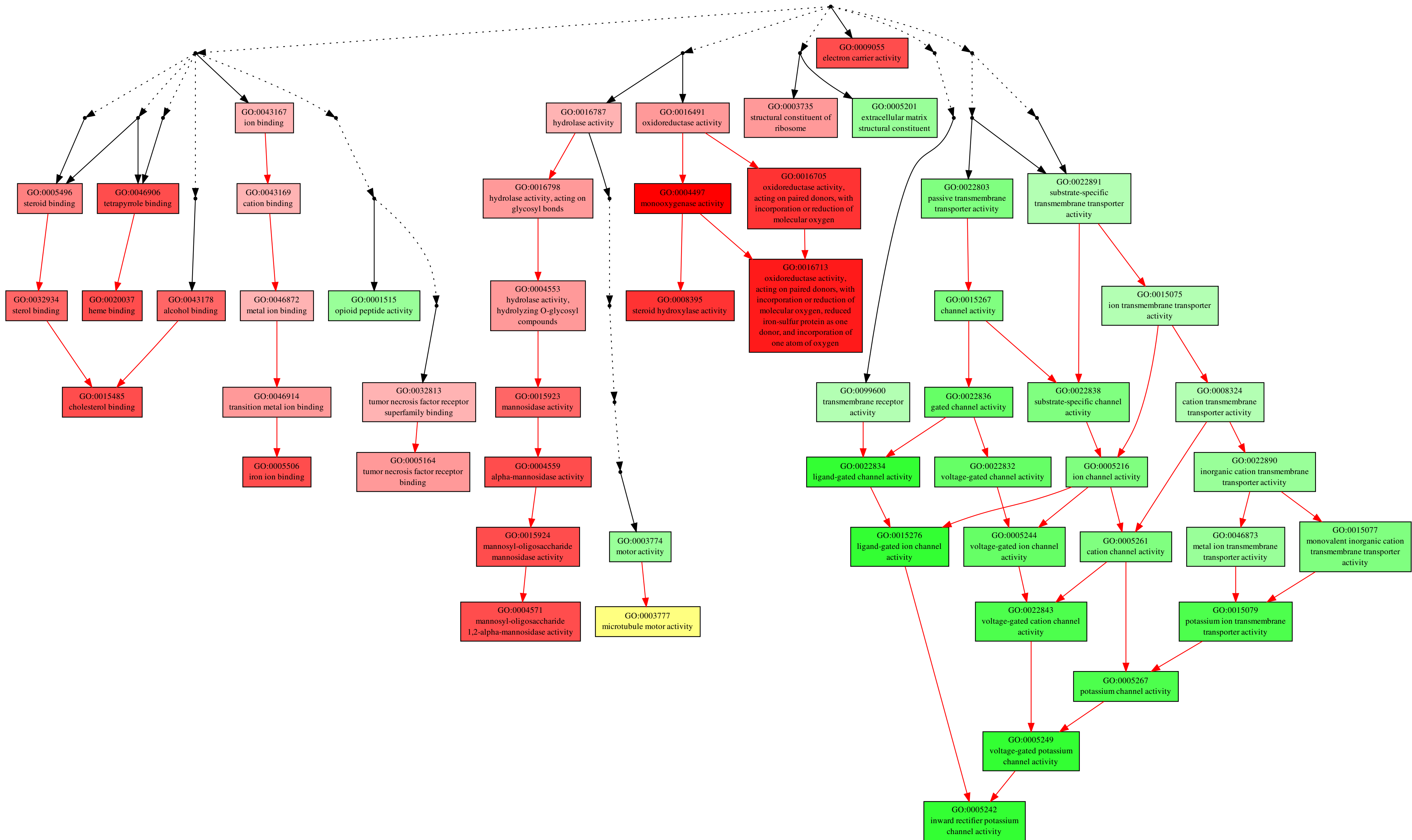

Supplement: Supplementary file 5 — Graphical output of Multi-GOEAST analysis results of Molecular Function GO terms in the top selected genes in CNA and RNA-Seq. (PDF 58 kb) [file 12920_2018_388_MOESM5_ESM.pdf]
